# A Versatile Method for Synthesis of Light-Activated, Magnet-Steerable Organic–Inorganic Hybrid Active Colloids

**DOI:** 10.3390/molecules28073048

**Published:** 2023-03-29

**Authors:** Dejia Geng, Lei Chen, Sinan Du, Xiang Yang, Huaguang Wang, Zexin Zhang

**Affiliations:** 1 College of Chemistry, Chemical Engineering and Materials Science, Soochow University, Suzhou 215123, China; 2 Centre for Soft Condensed Matter Physics and Interdisciplinary Research, Institute for Advanced Study, Soochow University, Suzhou 215006, China

**Keywords:** active colloids, polymeric colloids, wetting effects

## Abstract

The immense potential of active colloids in practical applications and fundamental research calls for an efficient method to synthesize active colloids of high uniformity. Herein, a facile method is reported to synthesize uniform organic–inorganic hybrid active colloids based on the wetting effect of polystyrene (PS) with photoresponsive inorganic nanoparticles in a tetrahydrofuran/water mixture. The results show that a range of dimer active colloids can be produced by using different inorganic components, such as AgCl, ZnO, TiO_2_, and Fe_2_O_3_ nanoparticles. Moreover, the strategy provides a simple way to prepare dual-drive active colloids by a rational selection of the starting organic materials, such as magnetic PS particles that result in light and magnet dual-drive active colloids. The motions of these active colloids are quantified, and well-controlled movements are demonstrated.

## 1. Introduction

Active colloids are synthetic microparticles that can convert various forms of energy, such as light [1,2,3,4,5], chemical [6,7], magnetic [8,9], electrical [10,11], and acoustic energy [12] into mechanical motion. Active colloids are found many applications at the microscale, including cargo delivery [5,13,14], biosensing [15], pollution monitoring [16,17], and environmental remediation [18,19], as well as acting as model systems to study the physics of active matter [20,21]. Holding the key to the success of active colloids in both applications and fundamental research is an efficient method to synthesize active colloids of high uniformity in large quantities. A synthetic active colloid is typically asymmetric and consists of two parts, an active part to act as the engine, and an inert part to break the symmetry and promote efficient directional motion. To synthesize active colloids, one can introduce such asymmetry by physical deposition or electrodeposition of metals. The resulting active colloids include spherical Janus active colloids [22,23] and bimetallic active colloids [24,25]. An alternative method for breaking symmetry is the formation of a dimer consisting of two particles with distinct properties. For example, by shielding electrostatic repulsive forces between nanoparticles of different metallic materials, nanoparticle aggregates, and dimer active colloids, together with various undesirable aggregations, can be produced [26]. Despite these methods, the synthesis of active colloids is still hindered by the often-complicated procedures and limited by the choice of materials [27,28]. In addition, most of the above-mentioned methods produce active colloids only in a small quantity with a broad distribution of sizes and shapes, limiting their usefulness in either the study of active matter physics or practical applications where uniformity is always the key. Therefore, an efficient method to synthesize uniform active colloids is urgently needed.

In this paper, a versatile, wetting-based method to synthesize organic–inorganic dimer active colloids with uniform sizes and shapes is reported. Specifically, organic polymer particles are first softened by a good solvent based on the well-known plasticization effect and then brought in contact with light-responsive inorganic nanoparticles. Due to the wetting effect of the softened polymer on the nanoparticles, the organic and inorganic components fuse together to form light-activated dimer active colloids, with the inorganic particles acting as active colloid engines. By this “soften and fuse” method, a myriad of active colloids with common polymer latex, polystyrene (PS) microparticles, and various photoresponsive inorganic nanoparticles are successfully prepared. In addition, magnet and light dual-drive active colloids are achieved when using magnetic PS particles instead of plain PS particles as one of the building blocks. We quantify the motion of the active colloid by video analyses and particle tracking, and demonstrate that the speed and the direction of the motion can be well controlled. Our method has the potential of mass-producing active colloids of uniform size and shape because the synthesis is solution-based and can be easily scaled up.

## 2. Results and Discussion

**Figure 1:**
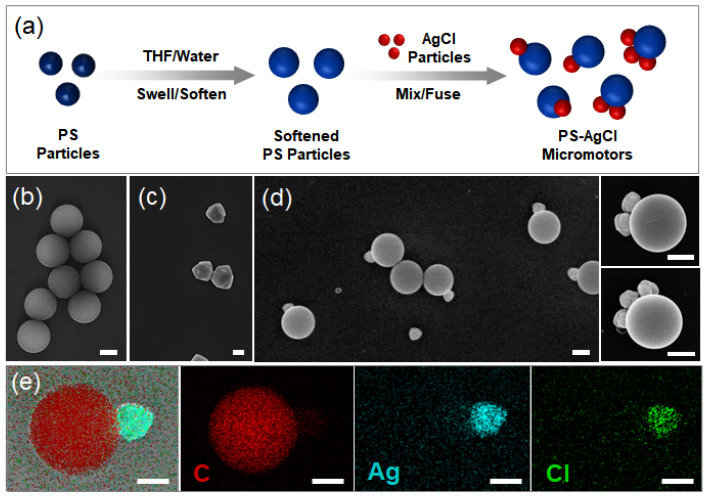
(**a**) Schematic of the synthesis procedure. PS particles (blue) are softened by tetrahydrofuran (THF) and then fused with inorganic particles, AgCl (red). (**b**–**d**) SEM images of PS particles, AgCl particles, and PS–AgCl hybrid dimer, trimer, and tetramer active colloids. (**e**) Energy-dispersive X-ray analysis of the C/Ag/Cl hybrid active colloids with an indication of the elemental composition. Scale bars: 500 nm.

Photoresponsive inorganic materials have been used to produce photoactivated active colloids such as shape-asymmetric AgCl particles under homogeneous UV irradiation [29,30] and TiO_2_ spheres under asymmetric light fields [26,31]. Here, a new strategy is proposed to produce organic–inorganic hybrid active colloids with asymmetries in both shape and chemical properties. The strategy is schematically illustrated in Figure 1a. The key step is the softening of the solid polymer (PS) particles with organic solvents. Our previous work has shown that PS particles can be progressively softened in a controlled manner when dispersed in a mixture of water and an organic solvent, such as THF or lutidine [31,32]. By varying the volume fraction of THF in the mixture, one can fine-tune the degree of softening (note that pure THF dissolves PS) [33]. When the softened PS particles are mixed with AgCl inorganic nanoparticles, the PS particles wet and fuse with the nanoparticles, and organic–inorganic hybrid active colloids are produced (Figure 1d). The fusing can be explained by the wetting effect of the polymer with the inorganic particles [34,35]. As a good solvent for polystyrene, THF can weaken polymer chain–chain interaction and strengthen solvent–polymer interaction. As a result, the solid polymer particle is plasticized, and becomes soft and sticky when suspended in the THF/water mixture. After AgCl nanoparticles are introduced into the mixture, the softened PS particles and the AgCl particles come into contact and fuse together. Thus, hybrid active colloids with the AgCl particles embedded into the PS particles are obtained, confirmed by energy-dispersive X-ray analysis (Figure 1e).

**Figure 2:**
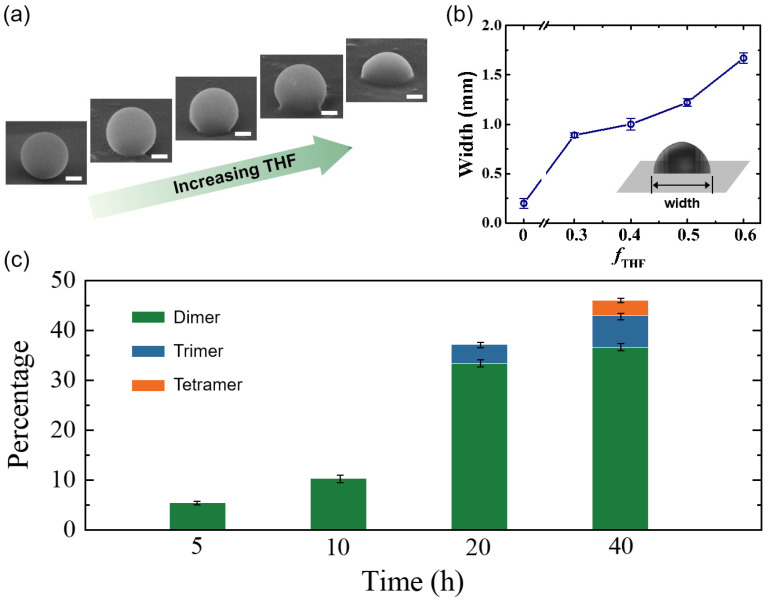
(**a**) SEM images of one PS particle on a silicon wafer after incubation in THF/water mixtures of different THF volume fractions: *f*_THF_ = 0.1, 0.3, 0.4, 0.5, and 0.6 (left to right). Scale bars: 500 nm. (**b**) Width of the wetting region of the PS particle with the wafer. The width increases with THF fractions. (**c**) The yield of PS–AgCl active colloids for different incubation times at *f*_THF_ = 0.4. The error bars in (**b**,**c**) are standard deviations and some error bars are smaller than the symbols in (**b**).

To verify the ‘soften and fuse’ mechanism, we examine and quantify the wetting process of the PS particle on an inorganic substrate, a silicon wafer. PS particles are dispersed in a mixture of THF and water with different volume fractions of THF, and the dispersion is tumbled for 24 h as for active colloid fabrication. We then observe the deformation and wetting of the PS particles on the wafer by SEM. Clearly, distinct states of particle deformation can be observed (Figure 2a). At low THF concentrations, there are no visible deformations of the PS particles. With increasing THF concentrations, significant deformations are observed, indicating the wetting of the PS particles with the silicon wafer. The degree of wetting was quantified by the width of the PS particles contacting the wafer (inset of Figure 2b). The width increases with the THF concentration, demonstrating stronger wetting ability at higher THF concentrations. Note that once the fraction of THF increases beyond 0.6, the PS particles tend to collapse after 24 h of incubation (Figure S3). Therefore, to enable wetting but prevent particle collapsing, an intermediate concentration, *f*_THF_ = 0.4, is selected. Moreover, it is found that the yield of the hybrid dimer active colloids increases with incubation time at *f*_THF_ = 0.4 (Figure 2c). However, the yield does not change significantly when the time is increased to 40 h, and longer incubation time only causes collapse and even dissolving of the PS particles. Therefore, we choose the optimal THF concentration, *f*_THF_ = 0.4, and the optimal incubation time, *t*_I_ = 24 h, to consistently and efficiently produce the organic–inorganic hybrid active colloids. Note that because the number of inorganic particles is in excess, one PS particle may fuse with more than one inorganic particle at a long incubation time (Figure 1d). However, dimers are dominant in all cases. The reason why only one inorganic nanoparticle is fused with the PS particle is suggested to originate from the surrounding flow field of the PS particle during the incubation. Once a small inorganic particle is embedded into the PS particle, the surrounding laminar flow field is disturbed [28], and thus other inorganic particles are difficult to approach and attach to the dimer particle. This results in mainly dimer particles instead of multimers. This change in the flow field is confirmed by numerical simulations (Figure S4).

**Figure 3:**
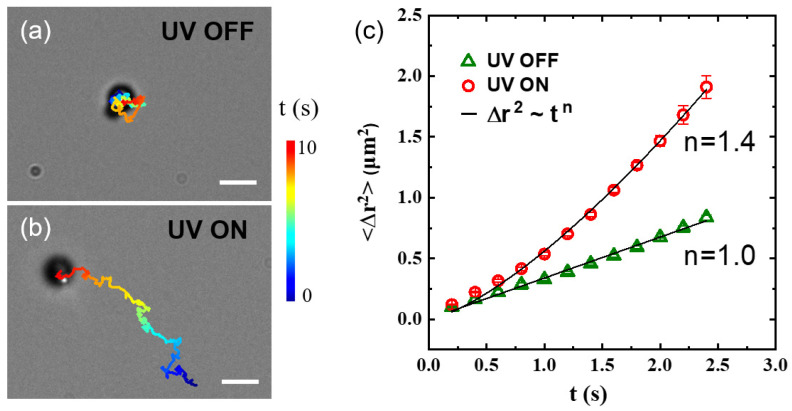
(**a**,**b**) Trajectories of PS–AgCl hybrid dimer active colloids in water upon turning the UV light (**a**) off and (**b**) on. The total time, shown color-coded, is 10 s. Scale bars: 2 μm. (**c**) Mean squared displacements as a function of lag time for the particles in (**a**,**b**). Solid lines are power-law fittings, with *n* = 1 indicating Brownian motion for a diffusive microparticle, and *n* = 1.4 > 1 indicating the super-diffusive motion of active colloid. The error bars represent standard deviations.

Due to the light-responsive inorganic nanoparticles, the as-prepared organic–inorganic hybrid particles can be activated by UV light and become active colloids. To demonstrate this, the motion of the PS–AgCl hybrid dimer is examined by bright-field optical microscopy. Without UV illumination, the particle undergoes Brownian motion similar to a typical colloidal particle (Figure 3a). However, as the UV light is switched on, an active, translational motion is observed (Figure 3b, see also Video S1). These two types of motions are quantitatively characterized by mean square displacement (MSD). The MSD for microscopic motion typically can be fitted to a general form, ${MSD\sim t}^{n}$. For Brownian motion, *n* = 1, while for active motion, such as the motion of active colloid studied here, *n* > 1. In fact, *n* = 1.4 for the PS–AgCl active colloid, signifying a super-diffusive motion (Figure 3b). Previous work on pristine AgCl active colloids has demonstrated that such fast motion results from the light-induced ionic self-diffusiophoresis of AgCl under UV illumination [28]. Specifically, when exposed to UV light, the AgCl component starts to decompose into protons and chloride ions. The protons diffuse much faster than the chloride ions (D_H_^+^ = 9.311 × 10^−5^ cm^2^·s^−1^, D_Cl_^−^ = 1.385 × 10^−5^ cm^2^·s^−1^) [36]. For the PS–AgCl active colloid, the different diffusion rates of protons and chloride ions result in a net electric field, which activates the fast motion of the active colloid.

**Figure 4:**
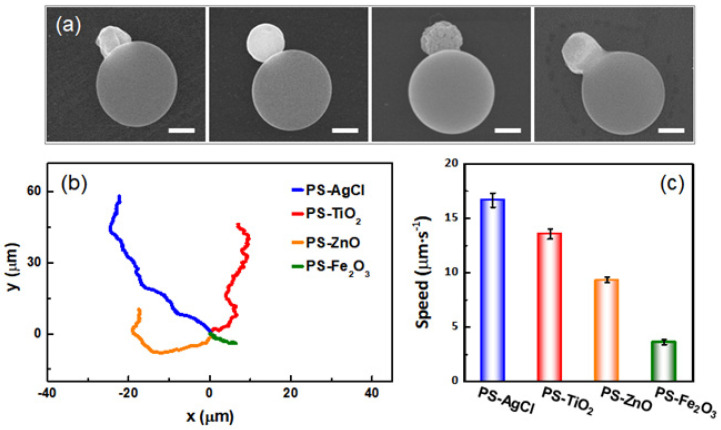
(**a**) SEM images of different hybrid dimer active colloids. The small inorganic particles embedded into the large PS particles are AgCl, TiO_2_, ZnO, and Fe_2_O_3_ (left to right). Scale bars: 500 nm. (**b**) The corresponding trajectories of the active colloids in 5 s under UV illumination and in 1% (*v*/*v*) H_2_O_2_ solution. The starting points of the trajectories are shifted to the origin for clarity. (**c**) Average speeds of different active colloids. The error bars represent the standard deviations of 10 measurements.

To illustrate the generality of our method, other types of organic–inorganic hybrid dimer active colloids are also prepared by using nanoparticles of different inorganic materials, including titanium dioxide (TiO_2_), zinc oxide (ZnO), and hematite (Fe_2_O_3_) (Figure 4a). XRD spectra demonstrate that the synthesis does not change the crystalline structures of the inorganic materials (Figure S2), and EDX spectra verify the successful preparation of the organic–inorganic hybrid dimer active colloids with these inorganic nanoparticles (Figure S5). All the active colloids are uniform in size because the starting particles, PS microspheres, and the inorganic particles are monodisperse (Figure S1 and Tables S1 and S2). This is advantageous for using active colloids as physical model systems in which monodispersity simplifies interpretation, and affords study of the condensed phases of many particles. To characterize the motion of these active colloids, the trajectory of each active colloid is shown in Figure 4b. By examining the contour lengths of the trajectories, one can infer that the speed of each type of active colloid is different. This observation is further confirmed and quantified by tracking the active colloids and calculating their speeds (Figure 4c). All four types of dimer active colloids move under the light because they each contain a photoactive material that under illumination generates a chemical gradient. In the case of AgCl and ZnO, it is likely that it is the diffusion of charged species (H^+^ and Cl^−^ for AgCl, Zn^2+,^ and OH^−^ for ZnO) that gives rise to ionic self-diffusiophoresis [28,37]. Active colloids containing TiO_2_ and Fe_2_O_3_ are well documented but less understood. Nevertheless, it is reasonable to assume the asymmetric chemical gradient generated by the photoactive lobe of the dimer creates a slip velocity on its surface and propels the TiO_2_ or Fe_2_O_3_ dimer active colloids (i.e., self-diffusiophoresis) [38,39]. There are a number of possible reasons for the speed differences observed among the four active colloids, such as a difference in their photocatalytic performance or zeta potential. These results demonstrate that our approach is universal in synthesizing different types of hybrid active colloids. This simple and versatile ‘soften and fuse’ strategy not only retains the unique properties of the organic polymer and inorganic nanoparticles, but also combines the advantages of the two materials, and makes those hybrid active colloids more attractive as nanomaterials than their single-component counterparts.

**Figure 5:**
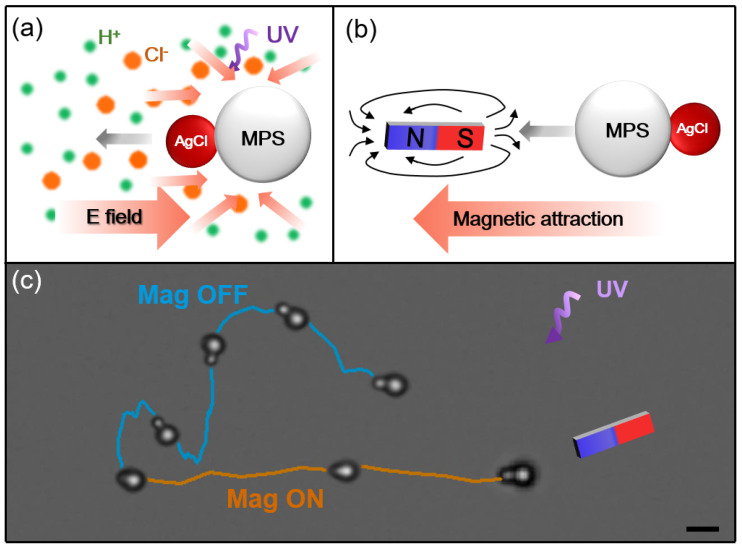
(**a**,**b**) Schematic diagrams showing the light-activated motion due to the AgCl particle, and the magnet-activated motion due to the magnetic PS particle (MPS). (**c**) Trajectory of MPS−AgCl particle moving under UV light (cyan line) and magnetic field (orange line). Superimposed time-lapse snapshots of active colloids demonstrate the orientation of the particles. Scale bar: 2 μm.

Similar to fuel and electricity dual-drive cars, dual-drive active colloids are more desirable than single-drive active colloids as they can utilize two different sorts of energy and harness a wider range of energy forms. The ‘soften and fuse’ method can be further exploited to prepare dual-drive active colloids. For example, instead of plain PS particles, magnetic PS particles (MPS, Fe_3_O_4_ core with PS shell) can be used to render hybrid active colloids with a magnetic response. As a result of the photoresponsive AgCl particles and magnetic-responsive Fe_3_O_4_ component, such active colloids can propel themselves and change direction by light and magnetic field. As illustrated in Figure 5, when UV light is applied, the AgCl component drives the active colloid to move by ionic self-diffusiophoresis [40], and the motion is toward the AgCl part and exhibits a fast random walk. When exposed to an external magnetic field by a neodymium magnet, the superparamagnetic Fe_3_O_4_ nanoparticles in the PS microparticles can be dragged by the magnet due to the magnetic field gradient, which is similar to the behavior of the magnetic active colloids reported recently [41,42]. In this case, the active colloid changes its orientation to align with the magnetic field and the direction of the motion is toward the magnet component. Now the MPS dominates the motion and the active colloid increases its speed more than threefold, from 16.1 μm·s^−1^ to 51.9 μm·s^−1^ (Video S2). Note that the magnet is set a little above the plane of the particle motion, and the active colloid rotates slightly, causing the view of the AgCl nanoparticle to be partly blocked by the large MPS sphere.

## 3. Materials and Methods

### 3.1. Materials

All chemicals (analytical grade) were obtained from Aladdin Bio-Chem (Shanghai, China) and were used as received without any further purification. All solutions were prepared with deionized water with resistivity ≥ 18.2 MΩ·cm^−1^.

### 3.2. Synthesis of PS/Inorganic Hybrid Active Colloids

The start materials for fabricating hybrid active colloids are common polystyrene (PS) spheres and inorganic particles including AgCl [43], TiO_2_ [44,45], ZnO [46], or Fe_2_O_3_ [47,48], all of which can be readily synthesized (see SI for their synthesis details and characterization). To prepare the hybrid active colloids, monodisperse PS particles were firstly swollen and softened by tetrahydrofuran (THF, a water-soluble plasticizer and a good solvent for PS). Next, 20 μL aqueous suspension of PS particles (40 mg·mL^−1^) was added to a glass bottle containing 1 mL mixture of THF and water (mixing ratio, 2:3 by volume). The mixture was stirred at room temperature for 30 min so that the PS particles became soft and sticky. Then 20 μL aqueous suspension of inorganic nanoparticles (80 mg·mL^−1^) was added to the mixture and the mixture was tumbled with an end-over-end motion at a speed of 60 rpm for 24 h at 25 C using a rotary incubator. The soft and sticky PS particles fused with the inorganic particles during the incubation. The resulting suspension was ‘quenched’ by dilution with a sufficient amount of water to solidify the PS particles; thus the PS–inorganic hybrid active colloids were obtained. The yield of the hybrid active colloids can be scaled up by increasing the amount of the solution (Figure S6).

### 3.3. Characterization of the PS/Inorganic Active Colloids

Scanning electron microscopy (SEM) images and energy-dispersive X-ray analysis (EDX) images were measured by Hitachi S-4700, while the X-ray diffraction (XRD) patterns of the samples were obtained on a Multipurpose X-ray diffractometer (X’Pert PRO MPD). The motion of the PS–inorganic hybrid active colloids was observed and recorded at 10 frames per second by a Basler ACE camera fitted on an Olympus IX73 microscope using a 40× objective. To prepare microscopy samples, active colloids were dispersed in H_2_O_2_ solutions (1% by volume) and the dispersion was loaded into a rectangular inspection chamber made of premium glass slides (Thermo Fisher, Waltham, MA, USA) and epoxy glue. The motion of the particles was triggered by a compact UV light with a wavelength of 365 nm and a maximum power intensity of 32 mW·cm^−2^.

### 3.4. Data Analysis

The active colloids in the micrographs were identified by image analysis using ImageJ (NIH) to obtain the particles’ positional coordinates. The positional data were further analyzed using in-house computer programs written in IDL (RSI) to calculate mean squared displacement and speed, and to plot the trajectories of the active colloids.

## 4. Conclusions

In summary, a versatile, solution-based strategy to synthesize organic–inorganic hybrid active colloids is reported. Our approach applies a known property of polymers, plasticization by organic solvent, into the advanced fabrication of active colloids. Our method is distinguished from other methods by several advantages, such as simple procedure, low cost, mass production potential, and more material options. Motion analyses confirm that the active colloids are self-propelled under UV illumination. Moreover, by rationally choosing the constituents of the hybrids, many types of active colloids can be achieved, indicating the generality of our method. Finally, the successful fabrication of a light and magnet dual-drive active colloid is illustrated. This simple, solution-based method opens up a new way to the scaled-up synthesis of active colloids, and will enable efficient production and diverse applications of active colloids.

## Data Availability

The data presented in this study are available on request from the corresponding author. The data are not publicly available due to privacy restrictions.

## Supplementary Materials

The following supporting information can be downloaded at: https://www.mdpi.com/article/10.3390/molecules28073048/s1. Figure S1: Scanning electron micrograph (SEM) of the organic polymer microparticles and inorganic nanoparticles; Figure S2: X-ray diffraction (XRD) pattern of the inorganic particles ((a) AgCl (b) ZnO (c) TiO_2_ (d) Fe_2_O_3_), and the corresponding hybrid active colloids; Figure S3: Bright-field optical micrograph showing PS particle collapse after incubation for 24 h in THF/water mixture; Figure S4: Schematic showing laminar flow near a spherical particle and turbulent flow near a non-spherical particle, and the numerical simulation; Figure S5: Energy-dispersive X-ray analysis of the hybrid active colloids with different nanoparticles; Figure S6: The production of the hybrid particles with ~1 L solution; Table S1: The sizes (mean, standard deviation) of the organic polymeric particle and the inorganic nanoparticles; Table S2: The lengths (mean, standard deviation) of the long axes of the dimer active colloids; Video S1: Motion of the PS–AgCl active colloid; and Video S2: Motion of the magnetic PS–AgCl dual-drive active colloid [49,50].
