# Supplementary material for: A Versatile Method for Synthesis of Light-Activated, Magnet-Steerable Organic–Inorganic Hybrid Active Colloids
Source: Molecules. 2023 Mar 29;28(7):3048. doi: 10.3390/molecules28073048 (PMC10095668; doi:10.3390/molecules28073048)
Supplement: Supplementary file 1 [file molecules-28-03048-s001.zip › molecules-2284649--SupplementaryMaterials.pdf]

Supplementary Materials

A Versatile Method for Synthesis of Light-Activated, Magnet-Steerable Organic–Inorganic Hybrid Active Colloids

Dejia Geng[1], Lei Chen[1], Sinan Du[2], Xiang Yang[2], Huaguang Wang[1, *], Zexin Zhang[1, 2,*]

[1]*College of Chemistry, Chemical Engineering and Materials Science, Soochow University, Suzhou 215123, China.*

[2]*Centre for Soft Condensed Matter Physics and Interdisciplinary Research, Institute for Advanced Study, Soochow University, Suzhou 215006, China.*

* Email: hgwang@suda.edu.cn (H. W.); zhangzx@suda.edu.cn (Z. Z.).

1. Supporting Videos

Video S1 Motion of the PS−AgCl active colloid. Without UV illumination, the active colloid undergoes Brownian motion and displays a random trajectory. As UV light is switched on, an active, translational motion is observed, and the active colloid exhibits a directional trajectory.

Video S2 Motion of the magnetic PS−AgCl dual-drive active colloid. Under UV illumination, the active colloid's direction of the motion is towards the AgCl part. When the magnetic field switches on, the active colloid increases its speed by more than three folds and changes its orientation to align with the magnetic field.

2. Supporting Figures and Tables

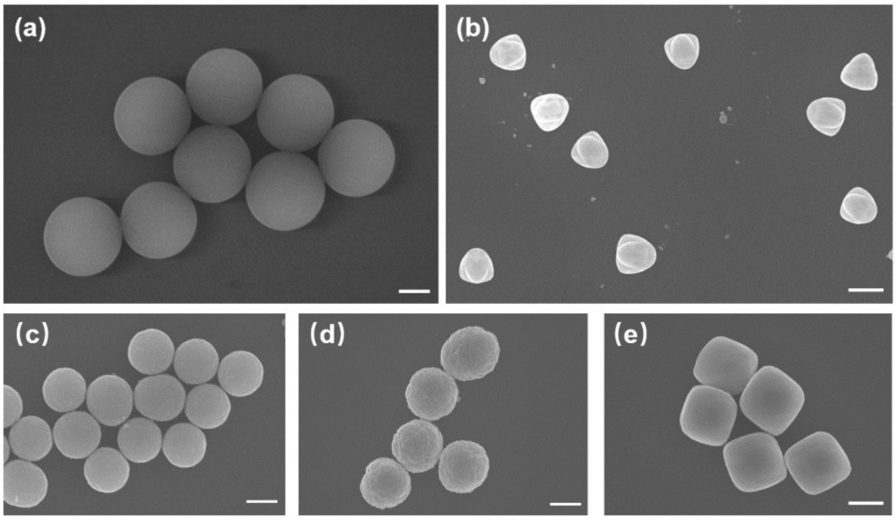

Figure S1. Scanning electron micrograph (SEM) of the organic polymer microparticles and inorganic nanoparticles: **(a)** PS **(b)** AgCl **(c)** TiO_2 **(d)** ZnO **(e)** Fe_2O_3. Scale bars: 500 nm.

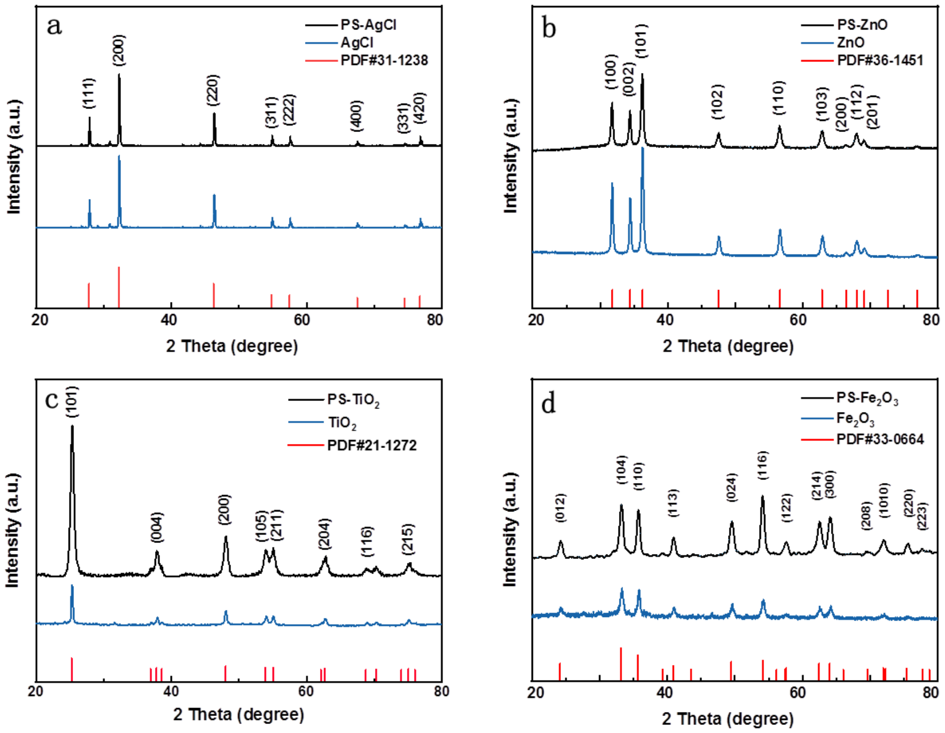

Figure S2. X-ray diffraction (XRD) pattern of the inorganic particles: **(a)** AgCl **(b)** ZnO **(c)** TiO_2 **(d)** Fe_2O_3, and the corresponding hybrid active colloids.

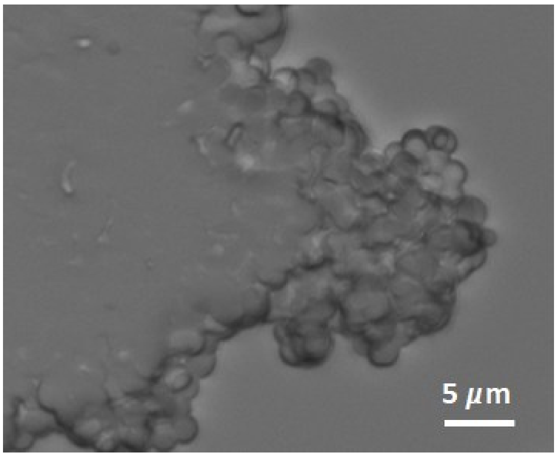

Figure S3. Bright-field optical micrograph showing PS particles collapse after incubation for 24 hours in THF/water mixture with THF concentration, f_{THF} = 0.65. Note that pure THF dissolves the PS particles completely.

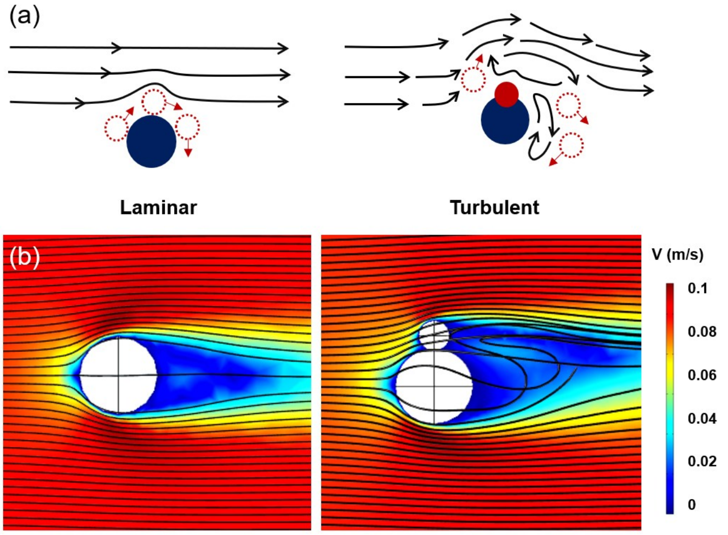

Figure S4. (a) Schematic showing laminar flow near a spherical particle and turbulent flow near a non-spherical particle. Dark blue and red spheres are the polymeric particle and the inorganic particle, respectively. Once a small inorganic particle is embedded into the PS particle, the surrounding laminar flow field is disturbed, and thus other inorganic particles are difficult to approach and attach to the dimer particle. **(b)** Numerical simulation indicates the flow field induced by a non-spherical particle is indeed turbulent (black lines), making it difficult for other particles to approach and contact the non-spherical particle.

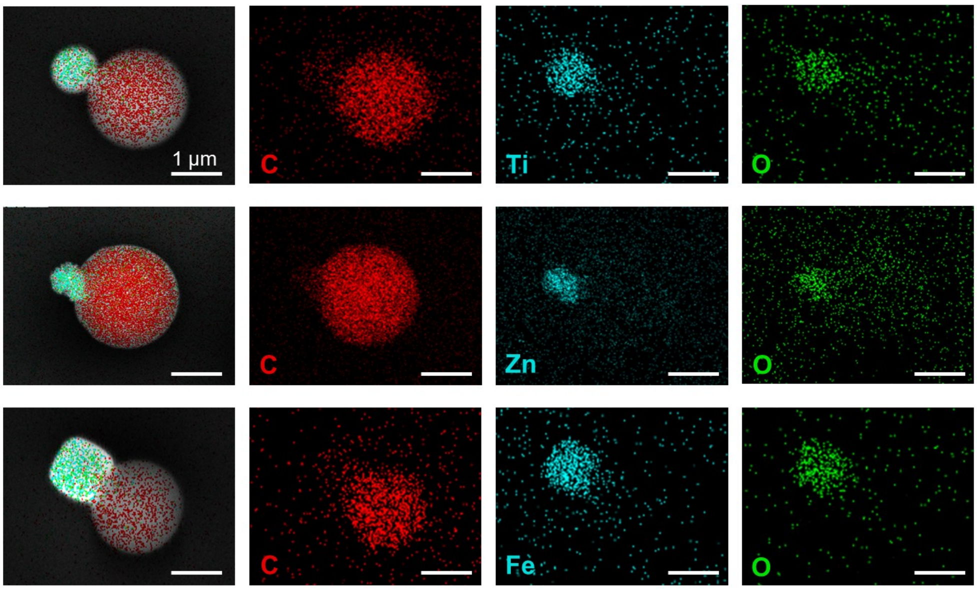

Figure S5. Energy-dispersive X-ray analysis of the hybrid active colloids with different nanoparticles: TiO_2 (top panel), ZnO (middle panel), and Fe_2O_3 (bottom panel). The elemental compositions are indicated. Scale bars: 1 μm.

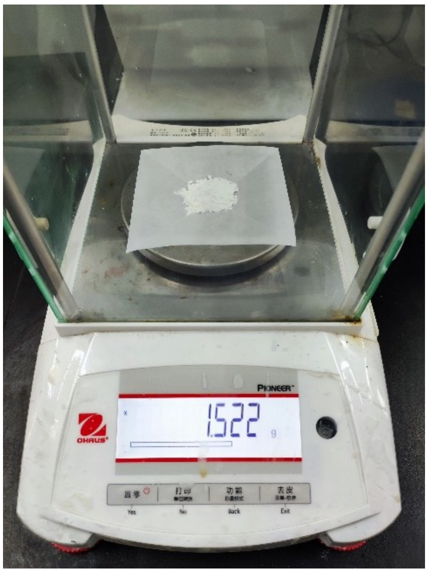

Figure S6. The production of the hybrid particles with ~1L solution.

**Particles**	**PS**	**AgCl**	**TiO_2**	**ZnO**	**Fe_2O_3**
**Size (μm)**	1.26 ± 0.03	0.57 ± 0.02	0.76 ± 0.03	0.86 ± 0.03	0.78 ± 0.02

Table S1. The sizes (mean, standard deviation) of the organic polymeric particle and the inorganic nanoparticles. Each of the polydispersity (mean/standard deviation) is less than 5%.

**Active colloidss**	**PS–AgCl**	**PS–TiO_2**	**PS–ZnO**	**PS–Fe_2O_3**
**Length (μm)**	1.69 ± 0.06	1.90 ± 0.07	1.98 ± 0.07	1.91 ± 0.05

Table S2. The lengths (mean, standard deviation) of the long axes of the dimer active colloids. Each of the polydispersity (mean/standard deviation) is less than 5%.

3. Materials and Methods

All chemicals (analytical grade) were obtained from Aladdin Bio-Chem (Shanghai) and were used as received without any further purification. All solutions were prepared with deionized water with resistivity ≥ 18.2 MΩ·cm^{-1}.

3.1 Synthesis of PS particles

In a typical synthesis, 10 ml styrene monomer was added to a 100 mL three-neck round-bottom flask containing 0.109 g of 2,2'-azobis(2-methylpropionitrile) (AIBN) as initiator. The mixture was set under the protection of nitrogen gas and emulsified by mechanically stirring at 500 rpm. In the other flask, 50.3 g anhydrous ethanol, 6 g deionized water, and 0.48 g polyvinyl pyrrolidone (PVP) as a stabilizer were mixed adequately under a nitrogen atmosphere for 30 min, and then the temperature was raised to 78 ℃. When this solution becomes turbid, the as-prepared styrene monomer and initiator mixture was slowly added by a peristaltic pump. After the addition was completed, the reaction continued for 8 hours and monodisperse PS particles were obtained. The particles were washed by centrifugation and redispersion in ethanol 5 times to remove any remaining reactant, and transferred to deionized water for further use [1, 2]. The SEM image of the PS particles is shown in Figure S1.

3.2 Synthesis of AgCl particles

Monodisperse AgCl particles were synthesized via the reaction of $AgNO_3$ and HCl in ethylene glycol in the presence of polyvinyl pyrrolidone (PVP) [3]. Firstly, 425 mg $AgNO_3$ and 415 mg PVP were dissolved in 50 mL of ethylene glycol. After mixing the solution with a magnetic stirrer for 20 min, 0.3 M HCl was added to the solution. The solution was then heated to 150 ℃and the reaction lasted undisturbed at this temperature for 6 h. After the solution was cooled down to room temperature, 100 mL of acetone and 150 mL of deionized water were added. The AgCl particles were cleaned by centrifugation and redispersion in deionized water 4 times. The SEM image and XRD graph of the AgCl particles are shown in Figure S1 and Figure S2.

3.3 Synthesis of TiO_2 particles

Monodisperse spherical TiO_2 particles were prepared by hydrolysis of titanium tetraethoxide ($Ti(OEt)_4$) in ethanol [4, 5]. First, 100 mL ethanol was mixed with 0 .4 mL salt solution (20 mM KCl) and 1.7 mL $Ti(OEt)_4$. The mixture was then stirred magnetically at room temperature under nitrogen atmosphere. The stirring was discontinued after a few minutes. After 2 hours the reaction was finished and the particles were collected by centrifuge and washed with ethanol. The final products were calcined in a tube furnace at 500 ℃ for 2 hours. The SEM image and XRD graph of the TiO_2 particles are shown in Figure S1 and Figure S2.

3.4 Synthesis of ZnO particles

Monodisperse ZnO microspheres have been successfully prepared by the hydrothermal method [6]. In a typical synthesis, zinc nitrate hexahydrate ($Zn\ (NO_3)_2 \cdot 6H_2O$) and triethanolamine (TEA) were used as zinc source and reaction solvent. First, 0.01 M Zinc salt mixed with 30 mL mixture (H_2O : TEA = 1 : 1) was put into a 50 mL flask and magnetically stirred. After 30 min, the solution was transferred into a Teflon reaction vessel. Then the tightly-sealed Teflon vessel was put in an oven maintained at 200 °C and reacted for 3 hours. The reaction was terminated by slowly cooling down the Teflon vessel to room temperature (25 °C). The white product from the reaction was collected by centrifuge and washing 4 times with deionized water and absolute ethanol, and finally dried at 60 °C for 6 h. The SEM image and XRD graph of the ZnO particles are shown in Figure S1 and Figure S2.

3.5 Synthesis of Fe_2O_3 particles

Monodisperse Fe_2O_3 hematite particles were prepared via the typical gel-sol process [7, 8]. The standard procedure is as follows: 40 mL of 2 M $FeCl_3 \cdot 6H_2O$, 30 ml of 6 M NaOH, and 5 mL of deionized water were stirred and mixed thoroughly. Then the solution was moved into a 100 mL Teflon reaction vessel. Then the tightly-sealed Teflon vessel was put in an oven maintained at 100 °C and reacted for 8 days. The final products were separated by centrifugation and washed repeatedly with deionized water. The SEM image and XRD graph of the Fe_2O_3 particles are showed Figure S1 and Figure S2.

3.6 Finite Element Modeling

To illustrate the flow field near the PS particles, finite element simulations were conducted with COMSOL Multiphysics package (version 5.2a). In the simulation, the dimer active colloid (one PS microsphere and one AgCl nanoparticle) is modeled as two touching spheres with no-slip boundary, and the fluid flows uniformly from a distance. Then we solved the Navier-Stokes equation for the flow field near the particle. In a typical simulation, more than 10^5 finite elements were used to ensure convergence.

3.7 Microscopic observation and video analysis

The motion of PS−inorganic hybrid active colloids was observed and recorded at 10 frames per second by a Basler ACE camera fitted on an Olympus IX73 microscope using a 40X objective. To prepare the microscopy sample, active colloids were dispersed in H_2O_2 solutions (1% by volume) and the dispersion was loaded in a rectangular inspection chamber made of premium glass slides (Thermo Fisher) and epoxy glue. The motion of the particle was triggered by a compact UV light with a wavelength of 365 nm and a maximum power intensity of 32 $mW \cdot cm^{-2}$. The active colloids in the micrographs were identified by image analysis using ImageJ (NIH) to obtain the particles’ positional coordinates. The positional data were further analyzed using in-house computer programs written in IDL (RSI) to calculate mean squared displacement and speed and to plot the trajectories of the active colloids.

References

[1] M. Okubo, M. Shiozaki, M. Tsujihiro, Y. Tsukuda, Colloid Polymer Science, 269 (**1991**) 222-226.

[2] H.R. Sheu, M.S. El-Aasser, J.W. Vanderhoff, Journal of Polymer Science Part A: Polymer Chemistry, 28 (**1990**) 629-651.

[3] S.-W. Kim, H.-E. Chung, J.-H. Kwon, H.-G. Yoon, W. Kim, Facile Synthesis of Silver Chloride Nanocubes and Their Derivatives, Bulletin of the Korean Chemical Society, 31 (**2010**) 2918-2922.

[4] S. Eiden-Assmann, J. Widoniak, G. Maret, Chemistry of Materials, 16 (**2004**) 6-11.

[5] K. He, G. Zhao, G. Han, Template-free synthesis of TiO2 microspheres with tunable particle size via a non-aqueous sol-gel process, CrystEngComm, 16 (**2014**) 7881-7884.

[6] H. Jiang, J. Hu, F. Gu, C. Li, Journal of Physical Chemistry, 112 (**2008**) 12138–12141

[7] T. Sugimoto, M.M. Khan, A. Muramatsu, Colloids and Surfaces A: Physicochemical and Engineering Aspects, 70 (**1993**) 167-169.

[8] M. Youssef, T. Hueckel, G.-R. Yi, S. Sacanna, Shape-shifting colloids via stimulated dewetting, Nature Communications, 7 (**2016**) 12216.
